# Incidence, mortality and survival in malignant pleural mesothelioma before and after asbestos in Denmark, Finland, Norway and Sweden

**DOI:** 10.1186/s12885-021-08913-2

**Published:** 2021-11-08

**Authors:** Kari Hemminki, Asta Försti, Tianhui Chen, Akseli Hemminki

**Affiliations:** 1 Biomedical Center, Faculty of Medicine and Biomedical Center in Pilsen, Charles University in Prague, 30605 Pilsen, Czech Republic; 2 Division of Cancer Epidemiology, German Cancer Research Center (DKFZ), Im Neuenheimer Feld 580, D-69120 Heidelberg, Germany; 3 Hopp Children’s Cancer Center (KiTZ), Heidelberg, Germany; 4 Division of Pediatric Neurooncology, German Cancer Research Center (DKFZ), German Cancer Consortium (DKTK), Heidelberg, Germany; 5 Department of Cancer Prevention/Zhejiang Cancer Institute, Cancer Hospital of the University of Chinese Academy of Sciences (Zhejiang Cancer Hospital), Institute of Basic Medicine and Cancer, Chinese Academy of Sciences, Hangzhou, 310022 China; 6 Cancer Gene Therapy Group, Translational Immunology Research Program, University of Helsinki, Helsinki, Finland; 7 Comprehensive Cancer Center, Helsinki University Hospital, Helsinki, Finland

**Keywords:** Incidence trends, Regional incidence, Risk factors, Age-specific incidence, Birth cohort analysis, Relative survival

## Abstract

**Background:**

Malignant pleural mesothelioma (MPM) is a rare but fatal cancer, which is largely caused by exposure to asbestos. Reliable information about the incidence of MPM prior the influence of asbestos is lacking. The nationwide regional incidence trends for MPM remain poorly characterized. We use nationwide MPM data for Denmark (DK), Finland (FI), Norway (NO) and Sweden (SE) to assess incidence, mortality and survival trends for MPM in these countries.

**Methods:**

We use the NORDCAN database for the analyses: incidence data were available from 1943 in DK, 1953 in FI and NO and 1958 in SE, through 2016. Survival data were available from 1967 through 2016. World standard population was used in age standardization.

**Results:**

The lowest incidence that we recorded for MPM was 0.02/100,000 for NO women and 0.05/100,000 for FI men in 1953–57, marking the incidence before the influence of asbestos. The highest rate of 1.9/100,000 was recorded for DK in 1997. Female incidence was much lower than male incidence. In each country, the male incidence trend for MPM culminated, first in SE around 1990. The regional incidence trends matched with earlier asbestos-related industrial activity, shipbuilding in FI and SE, cement manufacturing and shipbuilding in DK and seafaring in NO. Relative 1-year survival increased from about 20 to 50% but 5-year survival remained at or below 10%.

**Conclusion:**

In the Nordic countries, the male incidence trends for MPM climaxed and started to decrease, indicating that the prevention of exposure was beneficial. Survival in MPM has improved for both sexes but long-term survival remains dismal.

**Supplementary Information:**

The online version contains supplementary material available at 10.1186/s12885-021-08913-2.

## Introduction

Asbestos is probably the only known physical agent which is a dominant cause of a human cancer, in this case, malignant pleural mesothelioma (abbreviated here as MPM) [1]. Another unique feature of asbestos-related MPM is that the risk in exposed individuals does not appear to decrease after cessation of asbestos exposure [1, 2]. The estimated proportion of MPM caused by asbestos is at 80% or more [3–5]. Other possible causes or contributing factors include family history and related germline gene mutations (such BAP1 and BML), and exposure to erionite fibers in Turkey [6–10]. MPM is a fatal disease for which survival ranges between 4 and 12 months and for which unimodal or multimodal treatment is usually of little benefit [9]. We wanted to study epidemiology of MPM in the Nordic countries which are unique in starting nation-wide cancer registration before other countries, Denmark (DK) in 1943, Finland (FI) and Norway (NO) in 1953 and Sweden (SE) in 1958 [11]. Other relevant features from these countries include high-level medical care and essentially free-of-charge population access to medical care, which are relevant background data for interpreting population health outcomes.

Applications of asbestos started in the 1800s but a large-scale global use started around World War II (Robert L. Virta, Worldwide Asbestos Supply and Consumption Trends from 1900 through 2003. U.S. Geological Survey, Reston, Virginia: 2006, c1298.pdf (usgs.gov). The global annual production was 0.02 million (metric) tons in 1900, increasing to 1 million in 1950 and to 5 million around the peak year of 1975; by year 2000 it had declined to 2 million tons (Robert L. Virta, as above). By year 2000, the cumulative global production was estimated at 173 million tons. Much of asbestos still remains in the building materials and in environment (the produced amount accounts for some 35 kg/capita, assuming a global population of 5 billion). In the Nordic countries, the annual consumption in 1900 was 133 tons in DK, 24 tons in FI, 381 tons in NO and 336 tons in SE (Robert L. Virta, as above). In 1930, the consumption was somewhat over 1000 tons in each country. In 1950 it had increased to 9981, 9637, 2676 and 10,002 tons, and peaked around 1970 at 28,627, 12,035, 7962 and 18,646 tons when the respective populations were 4.9, 4.6, 3.9 and 8.0 million. By 1980 the consumption was about halved in DK and FI, essentially abolished in NO and dropped to 1200 tons in SE. At 1990 consumption was also abolished in FI but it remained at 800 and 600 tons in DK and SE, respectively. FI had asbestos mining until year 1975, producing the anthophyllite type of asbestos (in contrast to chrysotile accounting for over 90% of global mining); in total 350,000 tons were mined, of which 120,000 tons were used in Finland (Jorma Rantanen, Prevention and Management of Asbestos-Related Diseases in Finland, 252656_Asbesti.indd (julkari.fi).

We analyzed time trends in incidence, mortality and relative survival in MPM in the Nordic countries from their earliest available data until 2016. Because of the epidemic increase in the incidence of MPM after the 1960s in the industrial world, there has been a keen interest to see the culmination of the trend after the termination of asbestos use [12–16]. As the above asbestos consumption figures show, the major increase in the Nordic countries started after 1930 and the amounts increased 10-fold by 1950, (only 3-fold in NO) and a further 2–3-fold (less in FI) by the peak level at 1970. As the Nordic cancer registries started in the 1940s and 1950s we should be in the position of observing pre-asbestos influence, considering a lag time of around 20 years or more between the exposure and onset of MPM [9, 13, 17, 18]. Similarly, as the Nordic countries were among the first to ban asbestos use in the 1980s (FI 1992) (details in Methods), we have a chance to observe the impact of the bans to the MPM trends. We use the NORDCAN database, originating from the local cancer registries, in the present analyses.

## Materials and methods

### Regulation in asbestos use

The Nordic countries (DK, NO and SE) started to limit certain uses of asbestos with a resulting drop in imports in the 1970s. DK and NO banned the use of asbestos in the early 1980s, SE in 1986 and FI in 1992, but as one can see from the above consumption figure, DK and SE allowed some applications of asbestos (Asbestos bans around the world, (Asbestos bans around the world - Bainbridge (bainbridgeelearning.co.uk). The main uses of asbestos have been in cement (largest share by volume), insulation and pipefitting, car break linings and roofing materials. Accordingly, the occupational groups with the highest MPM risks have been workers in shipbuilding, construction and cement manufacturing, machine operation, seafaring and plumbing [7, 19–21]. However, the health hazards of asbestos are not over yet as asbestos containing insulation and building materials are in many cases still in place where deposited, awaiting removal. For example in Finland, a total of 300,000 tons of asbestos was estimated having been used and 2/3 remain in buildings and other structures in 1989/1990, 252656_Asbesti.indd (julkari.fi).

### Data analysis

We used the NORDCAN database, which is a compilation of data from the high-level Nordic cancer registries as described [22] (https://NORDCAN.iarc.fr/en/database#bloc2). In the database, MPM is covered by the codes C38.4, C45.0, C45.9 (i.e., cancer of the pleura, pleural mesothelioma, undefined mesothelial tumor, respectively). Incidence data were available from 1943 in DK, 1953 in FI and NO and 1958 in SE. Regional data from each country was available from a later starting date as shown in the figures. MPM-specific mortality data was available for FI from 1953 and for the other countries from a later date.

For incidence analysis, the world standard population was used in age adjustment. In incidence diagrams, generally 5-year smoothing was used because of small case numbers. As a consequence, in the figures showing incidence/mortality trends, the first and the last data point is at the third year from the start or from the end of the observation period.

Survival data for relative survival were available from 1967 onwards and the analysis was based on the cohort survival method for the first nine 5-year periods from 1964 to 2011, and a hybrid analysis combining period and cohort survival in the last period 2012–2016, as detailed [23, 24]. The hybrid method includes cases from the penultimate 5-year period to allow for a 5-year survival [25]. Age groups 0 to 89 were considered, and for age-standardization the International Cancer Survival Standard was used. The country-specific life tables were used to calculate the expected survival.

Aggregated data from a publically accessible database were used posing no ethical issues. Hence no ethical review applications were submitted.

## Results

Male MPM patient numbers ranged from 2065 (FI) to 3801 (SE) with median diagnostic age ranging from 67 (DK) to 71 years (NO and SE). Case numbers were lower for women, with a range from 357 (NO) to 1046 (DK), and median diagnostic ages somewhat higher, 70 (DK) to 73 years (FI and NO).

**Fig. 1:**
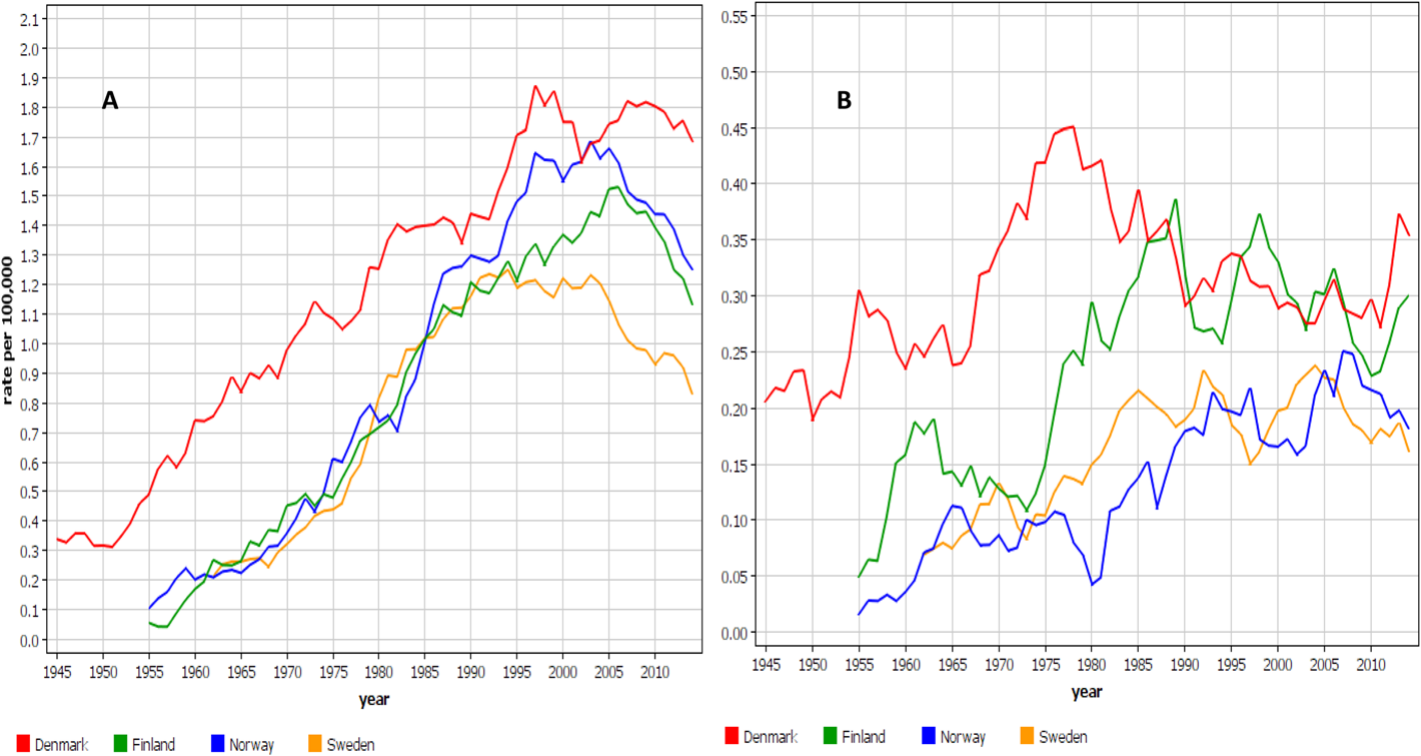
Age-standardized incidence trends for male (**A**) and female (**B**) pleural mesothelioma patients from Denmark, Finland, Norway and Sweden (5-year smoothing)

Age-standardized incidence rates for MPM in Nordic males are shown in Fig. 1A**.** The highest incidence was recorded for DK, which started at 0.3/100,000 in 1943–47 and reached a peak of 1.9/100,000 around 1997. The initial slope was linear and, after the peak incidence, the rate remained high. The rates for the other Nordic countries were superimposable in the exponential initial phase. The rates for FI started at 0.05/100,000 in 1953–57 and reached a maximum at 1.5/100,000 in 2006; for NO the start was at 0.1/100,000 and the peak was at 1.7/100,000 in 2003. By 2012–16 the NO incidence had dropped almost by a third. The rates for SE reached a broad maximum at 1.25/100,000 in the early 1990s and dropped by a third. The rates for females are shown in Fig. 1B. The starting incidence was somewhat lower than in men (NO 0.02/100,000) and the time-dependent increase was modest, reaching 0.45/100,000 for DK women in 1976. For FI women a maximum at 0.38/100,000 was reached in 1989. For NO and SE, the rates fluctuate at around 0.2/100,000 through the last three decenniums.

We compared incidence and mortality data for DK, FI and NO in Supplementary Fig. 1 (for SE MPM-specific mortality data were available only from 1998 onwards and not shown). For FI incidence and mortality data were available from 1953 onwards, and the mortality curve followed the incidence curve. This was also the case for the DK and NO data but periodically with larger gaps between the two curves, for unknown reasons.

**Fig. 2:**
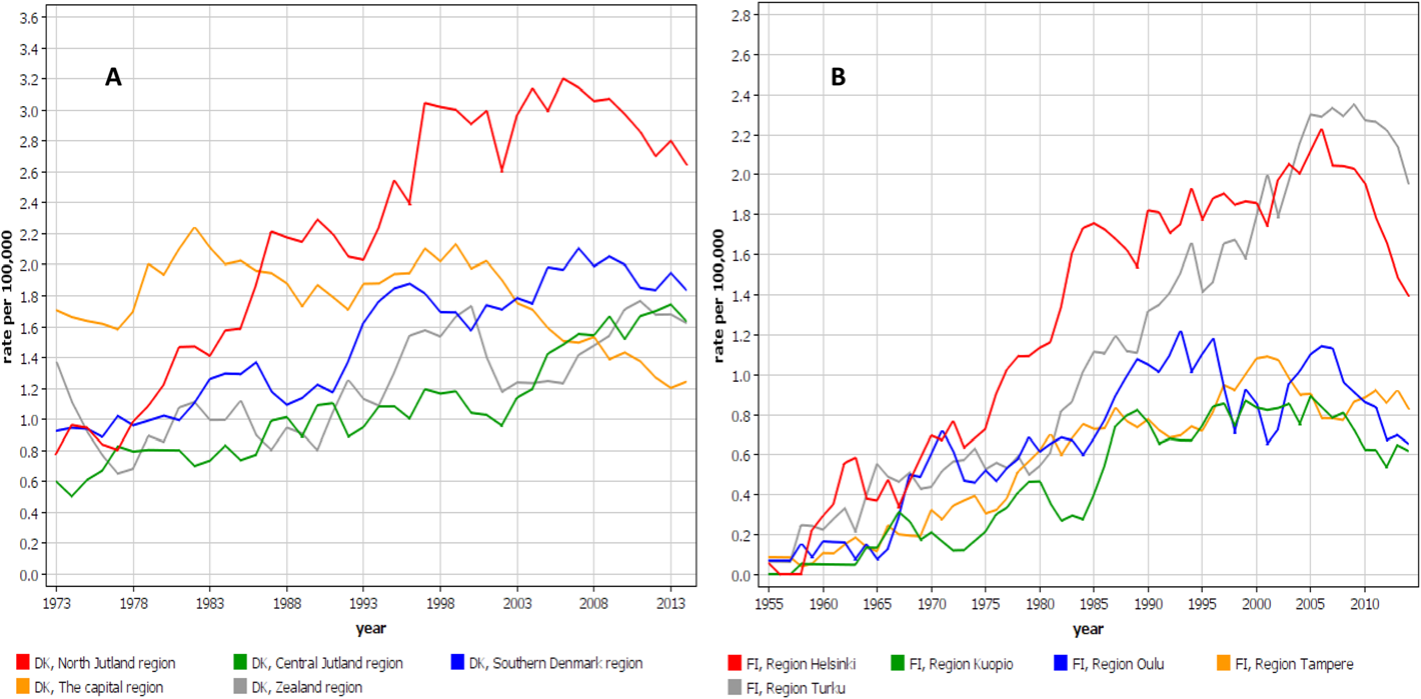
Age-standardized regional incidence trends for pleural mesothelioma from Denmark (**A**) and Finland (**B**) (5-year smoothing)

**Fig. 3:**
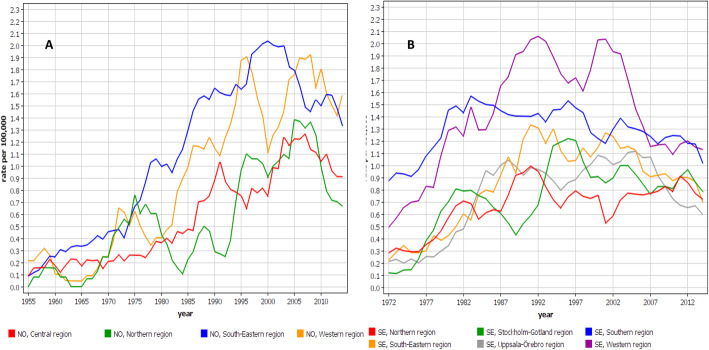
Age-standardized regional incidence trends for pleural mesothelioma from Norway (**A**) and Sweden (**B**) (5-year smoothing)

Incidence trends for DK and FI men are shown by region in Fig. 2. In DK the north Jutland region became very prominent in the course of time. The peak incidence at 3.2/100,000 in 2006 exceeded the lowest concurrent regional rate by almost 3-fold. In FI, the Helsinki region had initially the highest rate but it was taken over by the Turku region, reaching an incidence of 2.3/100,000 in 2005, and exceeding the lowest concurrent rates 3-fold. In Fig. 3 the trends are shown for NO and SE. In NO, the south-eastern region had the highest rates but towards the end of the observation period also the western region showed high rates. In SE, the southern region initially dominated but was taken over by the western region. In NO and SE the peak rates were about 2.0/100,000, and in NO the regional differences were smaller than in the other Nordic countries.

**Fig. 4:**
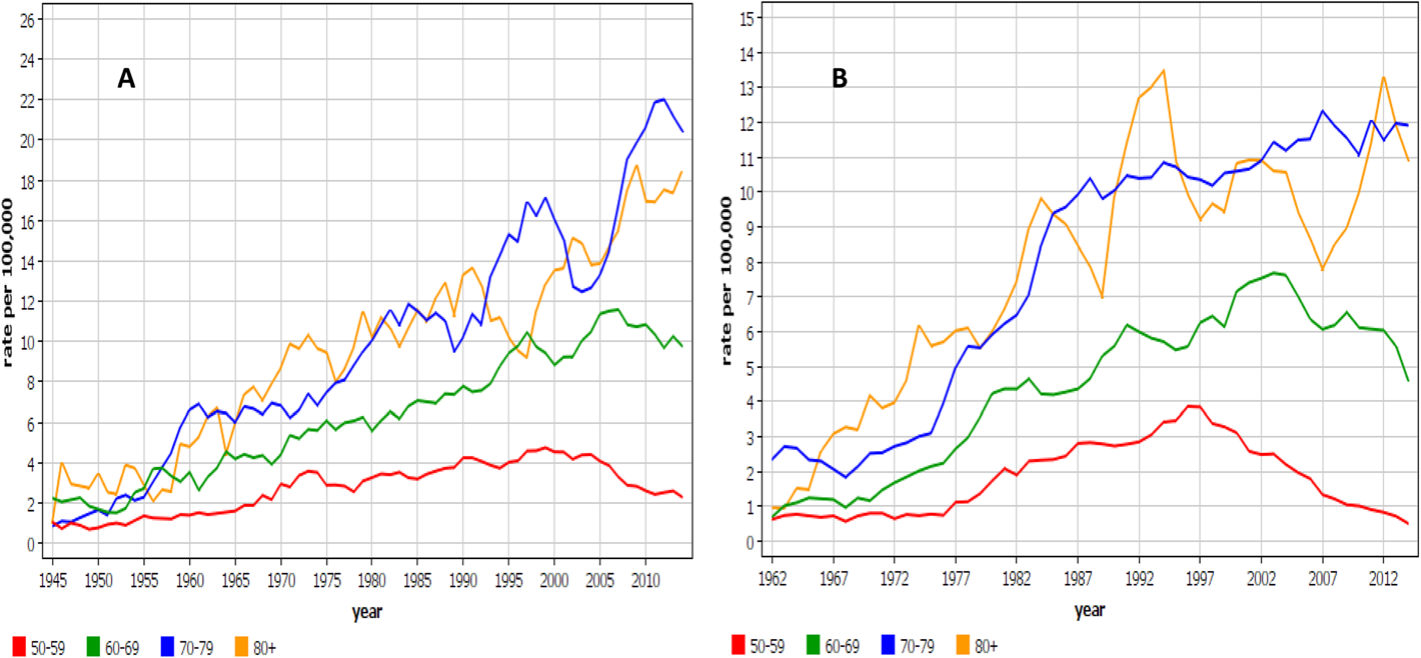
Incidence trends for pleural mesothelioma in Denmark (**A**) and Sweden (**B**) by age groups

Age-group specific analysis for male MPM showed that in DK the incidence rates increased in all but the youngest age groups throughout the observation period (Fig. 4A**)**. In SE the rates turned down in the two youngest age groups by year 2000, and in the two oldest ones the steep increase was broken into a modest increase in the late 1980s (Fig. 4B**)**. The FI and NO age-group specific incidence rates were similar, and between the DK and SE rates (Supplementary Fig. 2).

**Fig. 5:**
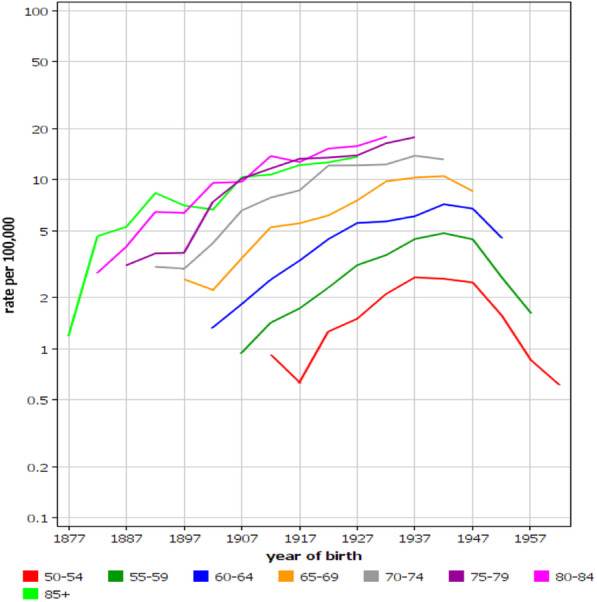
Pleural mesothelioma incidence trends for Nordic men (combining DK, FI, NO and SE) by 5-year birth cohorts. Note the logarithmic scale for the y-axis

Analysis by birth cohort in the combined Nordic male dataset (in order to expand the sample size) is shown in Fig. 5. For every birth cohort from 1877 onwards the age-specific incidence increased until birth cohorts 1937/1947; by then the increase leveled off and turned into decline. The incidence in MPM in birth cohorts 1907 and 1917 were almost equal in the three oldest diagnostic age-groups. As the y-axis is logarithmic, the implication is a strong increase in incidence at age 75–79 years in these birth cohorts. The changes by birth cohorts are clearer (albeit with fewer samples) in Supplementary Fig. 3A for Sweden and Supplementary Fig. 3B for the high-incidence western Sweden. The steep decline in incidence is apparent in the youngest birth cohorts. Similarly, the vastly increased risk in diagnostics age-groups 75–79 years in birth cohorts 1907 to 1937, and the later turning down of the incidence in that diagnostic age-group are evident.

**Fig. 6:**
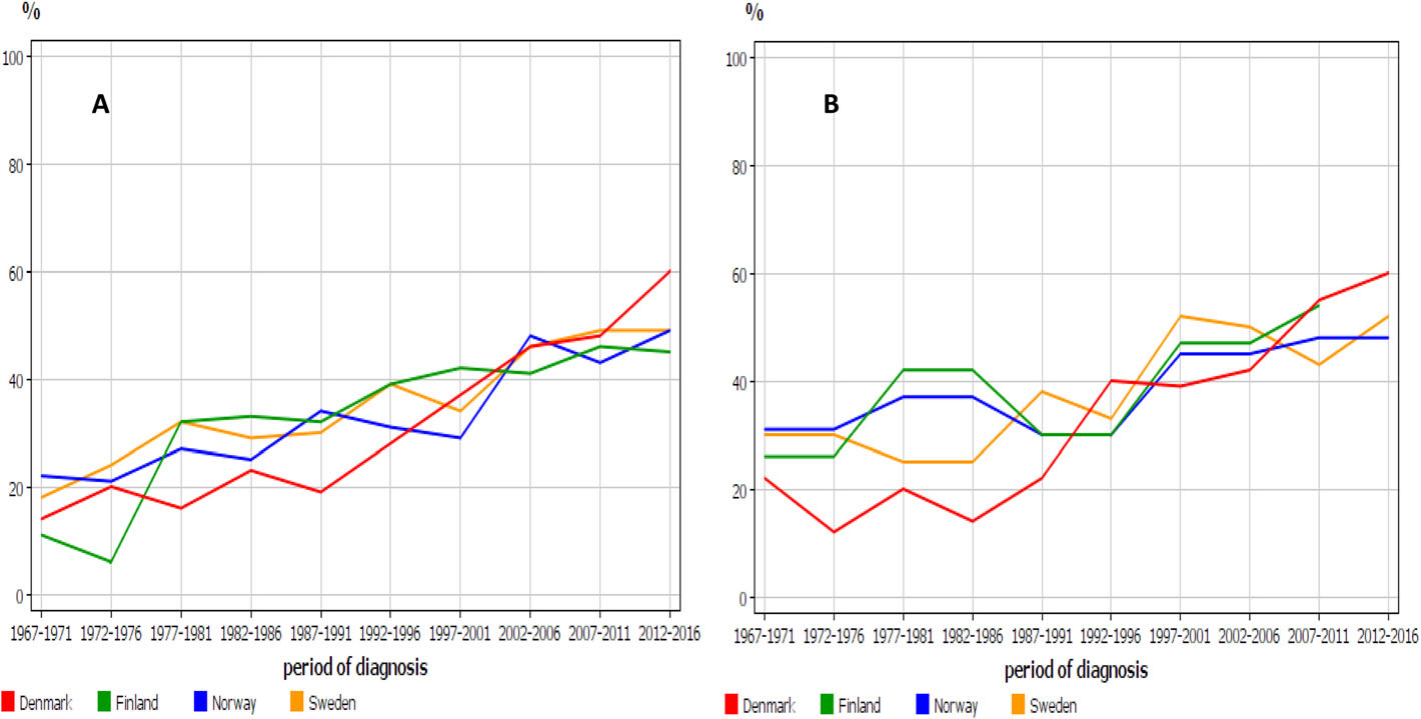
Relative 1-years survival for Nordic men (**A**) and women (**B**)

Relative 1-year survival is shown in Fig. 6 for men and women. Male survival was around 20% in 1967–71 it increased close to 50% in 2012–16. Survival for women was initially better than for men (20–30%) but the latest survival of about 50% was at the male level. No differences were observed between the countries. We analyzed also 5-year relative survival, which for women showed some fluctuation but for both sexes the latest survival was at or below 10% (Supplementary Fig. 4).

## Discussion

The novel findings of the present study were the estimates for the MPM incidence before essential influence of asbestos and the trends after its ban, for the likely period of maximal exposure and for the regional panorama of MPM epidemiology in the Nordic countries. We were able to confirm the poor survival in MPM, yet with a time-dependent improvement in the 1-year survival, probably due to earlier diagnosis, thanks to new imaging modalities [9]. Contributing to dismal 5-year survival, treatment options for MPM have been limited. Surgery can be performed only rarely, because the disease is typically diagnosed at an advanced stage. Radiotherapy is difficult because of the growth pattern of the disease along the pleural surfaces. Chemotherapy yields minimal benefits. Fortunately, immunotherapy has recently provided some hope for many MPM patients, but further advances are required to increase the number of patients benefiting [26–29]. Recognition of asbestos fibers as foreign by immune cells of the body may provide further avenues for development of immunotherapies in MPM [27].

The incidence in MPM was far lower in women than in men probably because exposures in women may be largely environmental rather than occupational [30, 31]. In order to have some confidence in the results we need to assume that MPM was reliably diagnosed; any new cancer type may be initially overlooked before the diagnostic criteria are set. While we can invoke the generally high quality of Nordic cancer registration, additionally the high concordance of the FI incidence (originating from the cancer registry) and mortality figures (originating from the cause of death register) adds to this confidence [11, 32]. This was also supported by the DK and NO data although with some inconsistencies. The main limitation is the ecological nature of the study and the rareness of MPM, resulting in large fluctuations in data points.

The lowest incidence that we recorded for MPM was 0.02/100,000 for NO women in 1953–57, which was not much lower than the rate for FI men at 0.05/100,000. The first Nordic ad hoc epidemiological study on MPM was published from the DK cancer registry reporting MPM rates of about 0.1/100,000 for men and 0.05/100,000 for women (European standard population) in 1943–47 [32]. These would be about 0.06 and 0.03/100,000 according to the world standard rate. These are at the level of the above FI and NO data but lower than our NORDCAN data for DK. In a recent study on global mesothelioma incidence (not separating the rare peritoneal mesothelioma) the lowest incidence of 0.14/100,000 was cited for East Asia in 1990 [33]. In that study, the highest rate of 2.26/100,000 was reported for Australia, which was only slightly higher than our highest rate of 1.9/100,000 for DK in 1997. Thus, the present study was able to gauge probably the most reliably measured lowest rates for MPM of around 0.02–0.05/100,000, and the DK peak incidence of 1.9/100,000, at the level of the highest national rates.

The analysis of regional MPM rates showed up to 3-fold intra-country differences, except less in NO. The DK peak in north Jutland at 3.2/100,000 is internationally high. The regional differences were telling about the sources of asbestos. In DK, north Jutland has been the center of cement manufacturing which has consumed 90% of the national asbestos [34]. Additionally the region has had shipbuilding industry [32]. In FI, Helsinki and Turku have been the centers of shipbuilding [30]. NO has traditionally been a seafaring nation and the home of half of all Nordic seafarers and 2/3 of all fishermen [35]. Seafarers have an increased risk of MPM (SIR 1.74) while fishermen have a reduced risk (0.43) [35]. This may explain the regional distribution of MPM in NO with highest incidence in the south-eastern part and lowest incidence in the northern parts where the fishing industry is concentrated to. The later increase in MPM in the western region in NO may be an indication of the North Sea oil and gas operations. In SE, the high regional incidence rates coincided with the main shipbuilding industries. In the western region, Gothenburg was one of the global centers of shipbuilding after World War II until its collapse in the 1970s. In the southern region (Malmö) cement industry probably boosted the initially high MPM incidence [21].

The incidence data by birth cohort may help to define the period of maximal MPM exposure. Figure 5 shows that in the birth cohort of 1907 the diagnostic age curves crossed suggesting that this birth cohort was exposed to something (asbestos) that pushed the MPM incidence at age 75–79 equally high as the incidence at higher ages. These men started to work around 1925. However, the age-specific incidence curves kept increasing up to birth cohort of 1937; these men started working around 1957 and their occupational history coincided with the peak period of asbestos use (1960–1975). The rapid decline in risk in birth cohort 1957 suggests that when these men started employment at around 1977 the worst exposures were already controlled. The data from the high-incidence western Sweden (Supplementary Fig. 3) show essentially the same pattern but with earlier initial incidence increases and sharper later decreases. The data suggest that the highest cumulative exposures in western Sweden was experienced by birth cohorts from 1907 through 1927.

How can we interpret the incidence differences and decline rates in terms of known consumption figures and occupational regulations? The present data are ecological and only allow speculations about the trends. In DK, the early rise and high incidence followed by slow leveling off would be consistent with an early and higher overall use of asbestos, exceptions to the occupational regulation and environmental contamination [31, 32]. The peaking of the female MPM incidence in the late 1970s may be related to early exposure and level of urbanization which was much higher than in other Nordic countries [32]. In FI, the incidence peaking in 2006 was later than in other countries, which agrees with the later ban on occupational use. In NO, the continued increase in incidence up to year 2003 may suggest that exposures of seamen are not easy to reduce (asbestos is in the ship interior) and this occupational group was dominant in NO. In SE, the dominant shipbuilding industry collapsed in the 1970s and simultaneously a major asbestos problem was solved. This helped SE to be among the first countries in the world to show the MPM incidence turn down [12, 16, 17]. In the same vein, the data help to predict the end of the asbestos problem (which is worse for men than for women). The SE data showed that the rates are relatively low and decreasing in age groups below 70 years. Thus, the MPM incidence may decline relatively fast as the exposed population dies/have died. For the other countries, the declines may be slower as only the age group below 60 years showed a clear declined in incidence. Finally, we have to remember that most of asbestos is still with us in the building materials (see Methods for uses of asbestos), and the third wave of asbestos disease caused by asbestos in place (buildings etc) will last [36].

In conclusion, MPM is an asbestos-related disease for which the latency from exposure to diagnosis is long and for which cessation of exposure may not reduce risk in exposed individuals [1, 2, 9]. In spite of limited exposures in medically alert countries, the use of asbestos continues in large volumes in many parts of the world, which predestines MPM as a global health problem for decenniums to come [16, 33, 37]. Although the global MPM incidence decreased from 1990 to 2017 from 0.52 to 0.44/100,000, the incidence increased in most countries [33]. We were able to trace the trends of MPM incidence from the era when asbestos influence was minimal to the era when it was maximal and showed the regional distributions in male MPM incidence, which matched the intensities of asbestos-related occupational activities. While we cannot predict when and if there might be an end to the asbestos-related MPM in the Nordic countries, the male incidence trends have turned down and survival in MPM has improved for men and women which suggests alleviation of the problem.

## Supplementary Information

**Additional file 1: Fig. S1.** Comparison of age-standardized male incidence and mortality trends for Danish (A), Finnish (B) and Norwegian (C) patients. The top (red) graph is incidence, the bottom one (green) is mortality. The scales for x-and y-axis differ between the countries. The widths of the diagrams are shown in proportion to the lengths of the observation period. **Fig. S2.** Incidence trends for pleural mesothelioma in Finland (A) and Norway (B) by age groups. **Fig. S3.** Pleural mesothelioma incidence trends for men from Sweden (A) and from the high-incidence western region (B) by birth cohorts. Note the logarithmic scale for the y-axis. **Fig. S4.** Relative 5-year survival for Nordic men (A) and women (B)

## Data Availability

Publically available NORDCAN data can be accessed at (https://NORDCAN.iarc.fr/en/database#bloc2).

## Abbreviations

BAP1: BRCA1 associated protein 1 gene
BML: recQ like helicase gene
DK: Denmark
FI: Finland
MPM: malignant pleural mesothelioma
NO: Norway
SE: Sweden
