# Supplementary material for: Incidence, mortality and survival in malignant pleural mesothelioma before and after asbestos in Denmark, Finland, Norway and Sweden
Source: BMC Cancer. 2021 Nov 8;21:1189. doi: 10.1186/s12885-021-08913-2 (PMC8576876; doi:10.1186/s12885-021-08913-2)
Supplement: Supplementary file 1 — Additional file 1: Fig. S1. Comparison of age-standardized male incidence and mortality trends for Danish (A), Finnish (B) and Norwegian (C) patients. The top (red) graph is incidence, the bottom one (green) is mortality. The scales for x-and y-axis differ between the countries. The widths of the diagrams are shown in proportion to the lengths of the observation period. Fig. S2. Incidence trends for pleural mesothelioma in Finland (A) and Norway (B) by age groups. Fig. S3. Pleural mesothelioma incidence trends for men from Sweden (A) and from the high-incidence western region (B) by birth cohorts. Note the logarithmic scale for the y-axis. Fig. S4. Relative 5-year survival for Nordic men (A) and women (B) [file 12885_2021_8913_MOESM1_ESM.pptx]

## Slide 1
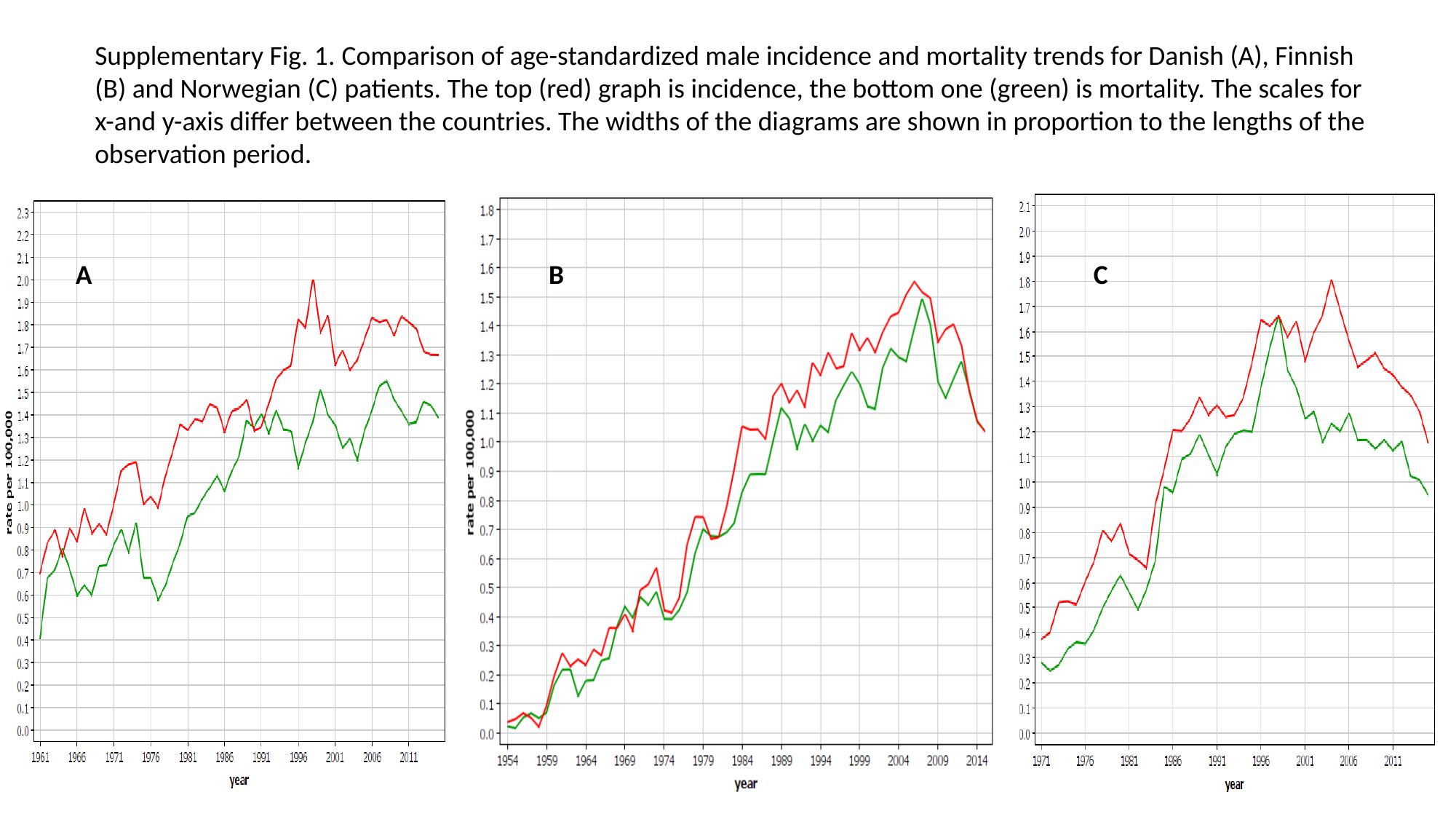

Supplementary Fig. 1. Comparison of age-standardized male incidence and mortality trends for Danish (A), Finnish (B) and Norwegian (C) patients. The top (red) graph is incidence, the bottom one (green) is mortality. The scales for x-and y-axis differ between the countries. The widths of the diagrams are shown in proportion to the lengths of the observation period.
A
B
C

## Slide 2
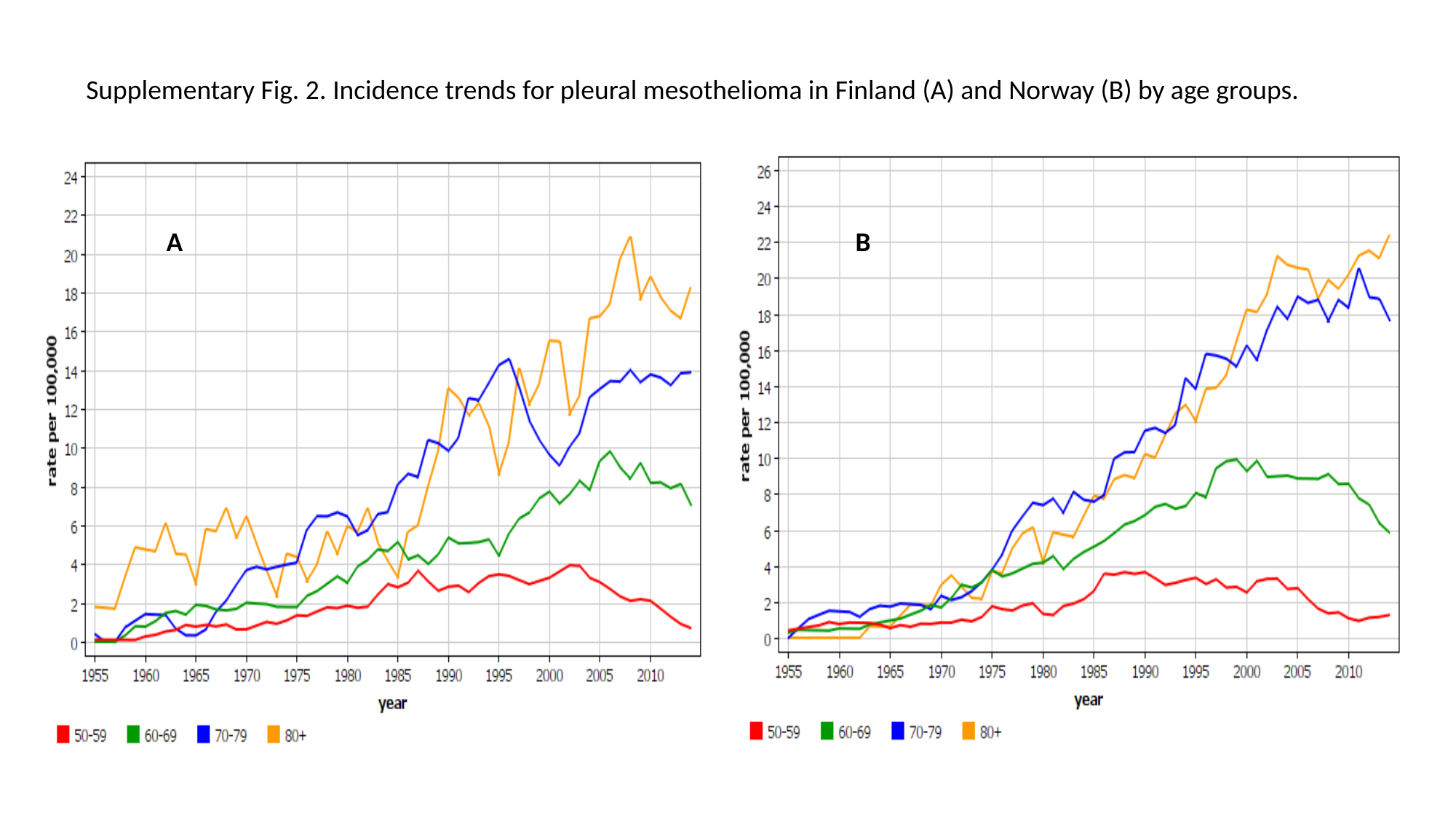

Supplementary Fig. 2. Incidence trends for pleural mesothelioma in Finland (A) and Norway (B) by age groups.
A
B

## Slide 3
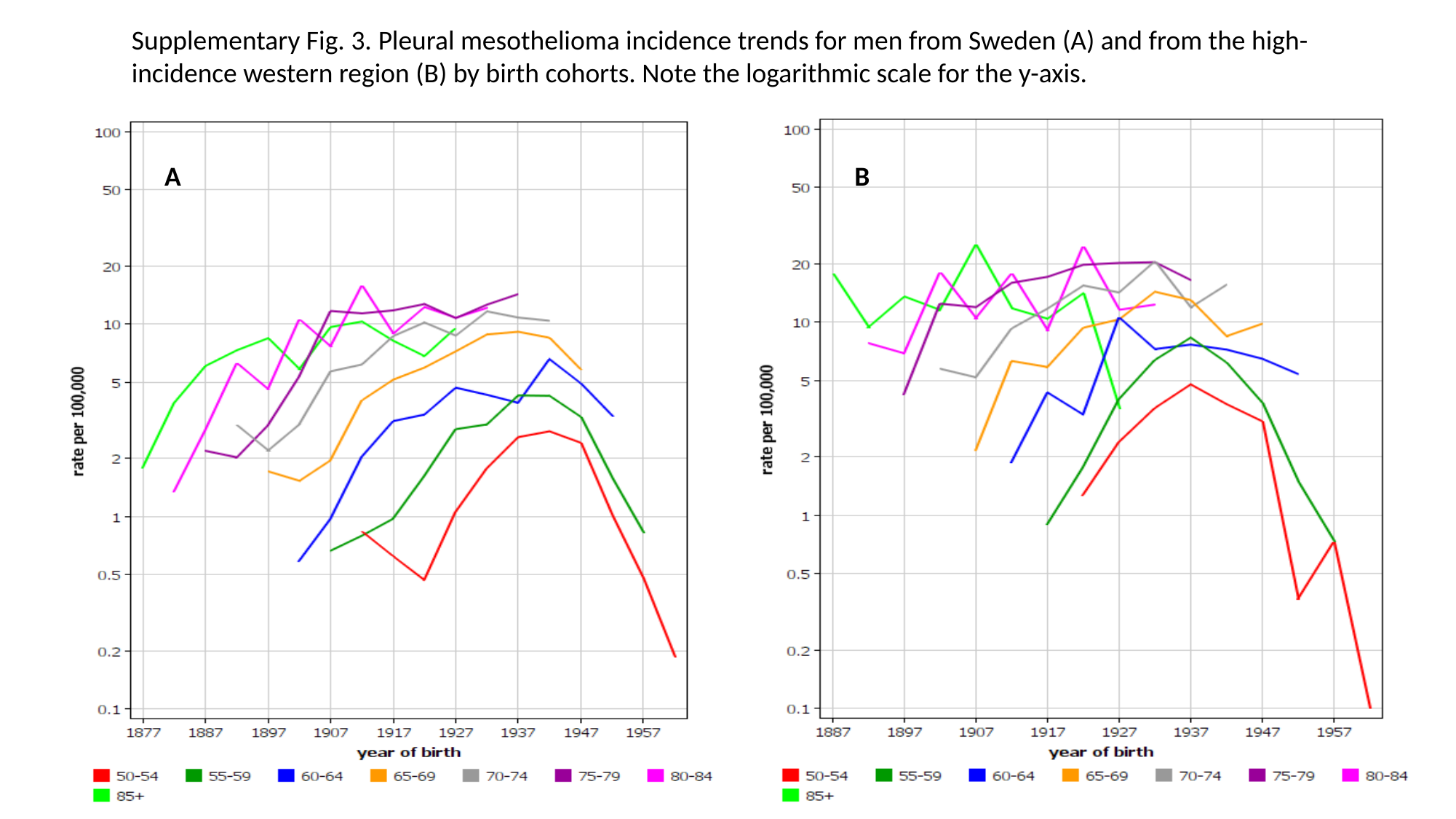

Supplementary Fig. 3. Pleural mesothelioma incidence trends for men from Sweden (A) and from the high-incidence western region (B) by birth cohorts. Note the logarithmic scale for the y-axis.
A
B

## Slide 4
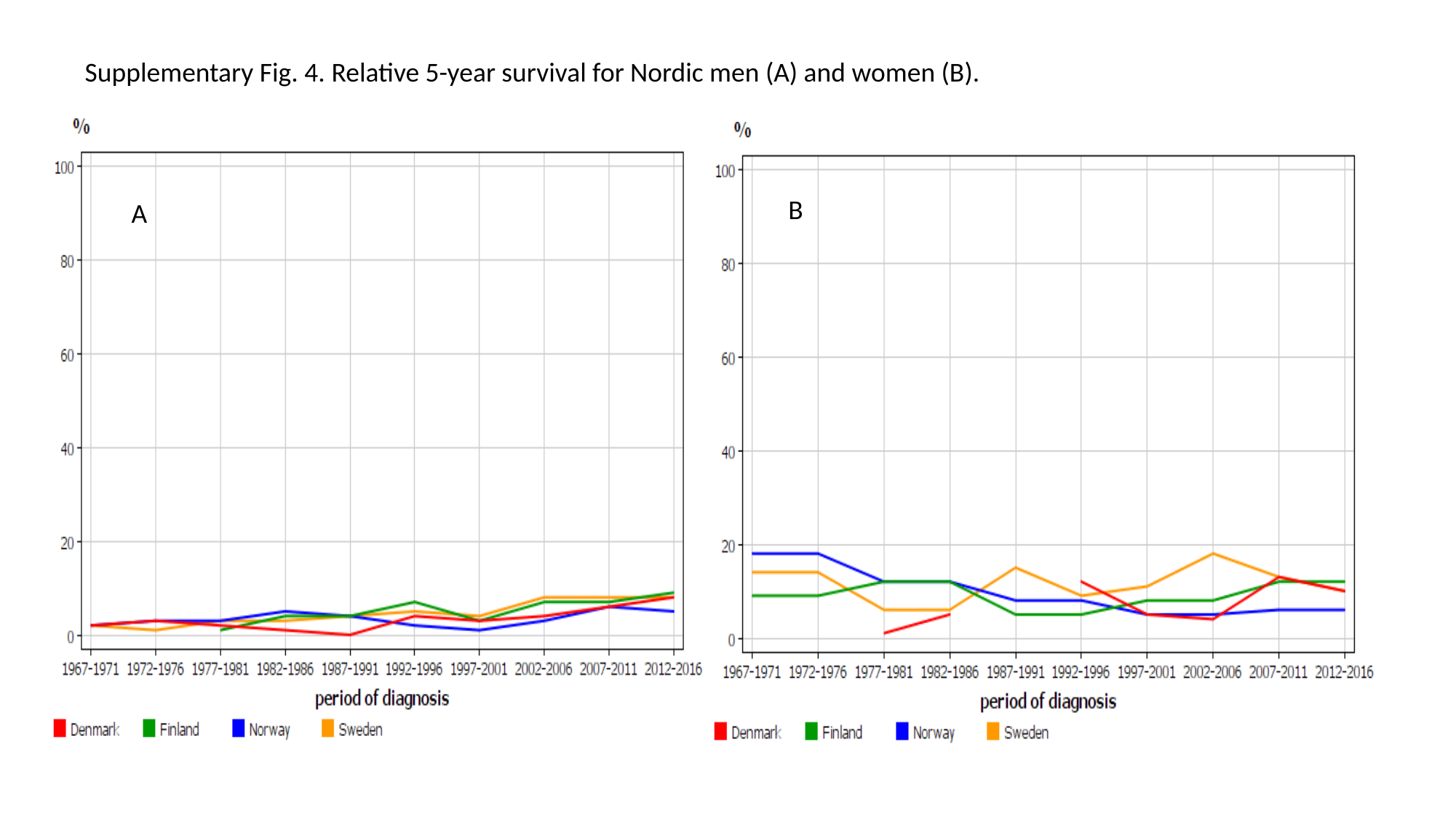

Supplementary Fig. 4. Relative 5-year survival for Nordic men (A) and women (B).
B
A
